# Association of emergency room admissions and weekdays in musculoskeletal medicine: results from a major trauma centre in Germany

**DOI:** 10.1186/s12873-025-01377-8

**Published:** 2025-11-04

**Authors:** Filippo Migliorini, Christian David Weber, Tommaso Bardazzi, Frank Hildebrand, Ulf Krister Hofmann

**Affiliations:** 1 Department of Trauma and Reconstructive Surgery, University Hospital of Halle, Martin-Luther University Halle-Wittenberg, Ernst-Grube-Street 40, 06097 Halle (Saale), Germany; 2 Department of Orthopaedic and Trauma Surgery, Academic Hospital of Bolzano (SABES-ASDAA), via Lorenz Böhler 5, 39100 Bolzano, Italy; 3 Department of Life Sciences, Health, and Health Professions, Link Campus University, Via del Casale di San Pio V, 00165 Rome, Italy; 4 Department of Orthopaedic, Trauma, and Reconstructive Surgery, RWTH University Hospital, Aachen, 52074 Germany; 5 Department of Orthopaedic, Trauma, and Reconstructive Surgery, Division of Arthroplasty and Tumour Surgery, RWTH University Hospital, 52074 Aachen, Germany

**Keywords:** Emergency room, Emergency department, Trauma, Musculoskeletal medicine, Orthopaedics, Weekday, Weekend

## Abstract

**Background:**

Seven days is a week. This ancient concept of structuring our everyday lives has survived several millennia. The repetitious cycle of work and rest is still shaping our routine, influencing the occurrence of diseases and necessities in emergency departments.

**Methods:**

We analysed the admissions to a trauma emergency department of a level 3 trauma centre from 2018 to 2024, looking for changes in admission frequency based on the seven days a week.

**Results:**

Data from 53,597 patients were collected, of whom 45.4% (24,336 of 53,597) were women. The mean age was 35.9 ± 25.4 years. A strong association emerged between the day of the week and the number of admitted patients. In particular, Saturday and Sunday had the most admittances, whereas Thursday was the least busy (*P* < 0.01).

**Conclusions:**

While we cannot present data on the reasons for this increase, it is probable to account for this rise in a different activity profile of the patients in comparison to the work week. Other factors that might influence this are the patients’ obligations during the work week and the availability of medical care limited to ERs on weekends. Independent of all these reasons, these data may help healthcare providers allocate their resources based on patient volume and emergency conditions.

**Supplementary Information:**

The online version contains supplementary material available at 10.1186/s12873-025-01377-8.

## Introduction

Seven days is a week. While the concept of a repetitive cycle of days forming a unified time unit has been present in many cultures throughout history, Judaism established its seven-day time course under Babylonian influence [1]. The concept of a week is a time unit that is not determined by any natural event and, as such, represents an arbitrary human category [1]. However, the length of the week is derived from the four phases of a lunar cycle, which takes approximately 28 days. From Judaism, the seven-day week was transmitted to Christianity, which in turn influenced the Roman Empire to adopt it officially in the year 321 CE, thus laying the foundation for its later spread across Europe and, much later, throughout the world. While the consequences of such a weekly routine differ between states and cultures, it is expected to involve a workweek with contrasting work activity on weekends. This can be Sunday in countries with Christian traditions or Saturday or Friday in Jewish or Arab cultures, respectively. Being such a global phenomenon marked by the contrast between the workweek and the weekend, it powerfully shapes our everyday rhythm and routine.

From a medical perspective, it is well established that the circadian system has a significant influence on disease severity. Adverse cardiovascular incidents, for example, peak in the morning, asthma appears worse at night, and temporal lobe epileptic seizures are most prevalent in the afternoon [2]. These observations have been attributed to circadian rhythms in physiology, such as cortisol levels or our sleep and wake patterns [2] and are, at their core, something that biology dictates. However, the week’s rhythm is a cultural habit without any internal physiologic clock driving it. Are accidents and onsets of disease thus related to the rhythm of the week? The easiest way to approach this question is to analyse data from emergency rooms (ERs), which are the gateway to triage and further specialised care in many healthcare systems. Several studies have already looked into this question on a broad basis in the past few years: Ebrahimihoor et al. (2023) or Karpman et al. (2021), for example, analysed the association of the day of the week and the arrivals in ERs in the US and found the highest number of admissions on Mondays and the lowest number of cases on the weekend [3, 4]. This is in line with the observation that for acute myocardial infarction, a septarian rhythm has been described with a peak on Mondays and the lowest rate on Saturdays [5–9]. This effect, however, appears to be small, and potential explanations range from an association with alcohol consumption to increased stress levels at the beginning of the week [5]. Regarding peptic ulcers, an inverse relationship was observed, with more patients attending the ER on weekends and during holidays in Taiwan [10]. Notably, the relative increase on weekends compared to weekdays in this study was significantly lower for patients with peptic ulcers who had bleeding or perforation than for those without these complications. Similarly, the relative increase on the weekend was also much lower in the population over 65 years, suggesting a strong dependency of the patient volume in the ER on patients’ everyday life obligations. Other conditions which have been repeatedly reported to have a weekly pattern are, for example, panic-attack admissions with a sharp rise on the weekend [11], acute diverticulitis with the highest values on the weekend [12], and both stroke [13, 14] and transient ischemic attacks with a peak on Mondays [15].

In the present study, we analysed admissions to a trauma emergency department of a level 3 trauma centre from 2018 to 2024, looking for changes in admission frequency based on the seven-day week.

## Methods

### Study design

The present study was conducted according to the principles of the Declaration of Helsinki and was approved by the ethics committee of the RWTH Aachen University (project ID EK 121/22). The present study follows the Strengthening the Reporting of Observational Studies in Epidemiology: the STROBE Statement [16]. The present investigation was conducted at the Department of Orthopaedics, Trauma and Reconstructive Surgery of the University Hospital RWTH Aachen, Germany. In April 2024, the clinical databases of the institutions were accessed. For the databases of the German institution, the OPS (operation and procedure codes) reported in Appendix 1 were used in combination with the ICD (International Statistical Classification of Diseases and Related Health Problems) codes, also noted in the appendix. All data from trauma patients admitted to this ED from 2018 to 2024 were retrieved. Patients’ sensitive information was pseudonimised, and data were included in a Microsoft Excel spreadsheet (version 16.6).

### Data collection

Two authors independently retrieved patients’ data. The following patients’ data were recorded: gender, age at admission, and admission date. The number of patients admitted on each day was registered.

### Statistical analysis

All statistical analyses were performed by the main author (FM) using the software STATA/MP 16.1 (StataCorp, College Station, TX). For each endpoint, the arithmetic mean and standard deviation were calculated. A Chi-squared test was performed to assess the significance of the association between the number of patients admitted to the emergency trauma department and the day of the week. Values of *P* < 0.05 were considered statistically significant.

## Results

### Patient demographics

The database search yielded 53,997 admissions to the ER between 2018 and 2024. There were no exclusions. Therefore, 53,597 patients were considered for the study. Data were collected from 53,597 patients, of whom 45.4% (24,336 of 53,597) were women. The mean age was 35.9 ± 25.4 years. Demographic data are summarised in Table 1.

**Table 1:** Patients’ demographics

Endpoint	Patients	Females	Mean age
Total	53,597	45.4% (24,326 of 53,597)	35.9 ± 25.4
Monday	7,491	45.7% (3,420 of 7,491)	36.1 ± 25.1
Tuesday	7,124	45.8% (3,260 of 7,124)	37.2 ± 25.6
Wednesday	7,235	45.8% (3,313 of 7,235)	35.5 ± 25.6
Thursday	6,941	46.1% (3,197 of 6,941)	35.8 ± 25.7
Friday	7,921	46.5% (3,682 of 7,921)	36.1 ± 25.5
Saturday	8,549	44.2% (3,778 of 8,549)	35.8 ± 25.2
Sunday	8,336	44.1% (3,676 of 8,336)	35.2 ± 24.9

### Result syntheses

The observed frequencies from the sample data differed significantly from a homogeneous distribution. Indeed, a strong correlation emerged between the day of the week and the number of admitted patients. In particular, Saturday and Sunday had the most admittances, whereas Thursday was the least busy (*P* < 0.01, Table 2).

**Table 2:** Admittances’ contingency table

Weekday	Expected patients	Observed patients	Expected proportion	Observed proportion	95% CI	*P*
Monday	7,657	7,491	14.3%	14.0%	13.7–14.3	0.04
Tuesday	7,657	7,124	14.3%	13.3%	13.0–13.6	< 0.01
Wednesday	7,657	7,235	14.3%	13.5%	13.2–13.8	< 0.01
Thursday	7,657	6,941	14.3%	13.0%	12.7–13.2	< 0.01
Friday	7,657	7,921	14.3%	14.8%	14.5–15.1	< 0.01
Saturday	7,657	8,549	14.3%	16.0%	15.6–16.3	< 0.01
Sunday	7,657	8,336	14.3%	15.6%	15.2–15.9	< 0.01
Total	53,597	53,597	100%	100%		

(CI: confidence interval)

## Discussion

In the present study, we analysed the weekdays of admissions to a trauma emergency department of a level 3 trauma centre for 2018–2024. The weekday patient load appears function of the medical condition that prompts the patient to attend the ER. Several other works have already addressed the question of trauma admissions in various contexts [17]. Consistent with our data, multiple studies have reported the highest trauma admission volume on weekends [18–21], with approximately one-third of patients being admitted on weekends, thereby exceeding the weekday average [21]. This observation even seems to hold for paediatric patients [22]. Of note, Tiruneh et al. also described a higher risk of hospitalisation from violence and fall-related injuries but a lower risk for admissions caused by road traffic accidents [21]. Giannoudis et al., 2016, also described that patients admitted to a Level I Major Trauma Centre in the UK for polytrauma were younger, more severely injured and presented with a higher incidence of haemodynamic instability [23]. These observations can be accounted for by a different activity profile on days off than during regular workdays, where most activities are embedded in a high routine and, for example, by workers’ compensation boards, optimised for injury prevention. Moreover, many jobs in Western countries have a sedentary profile, reducing the risk of acute trauma. In contrast, the weekend is often associated with physical activities that, in many instances, carry a higher potential for injury. Numerous articles dwell on the phenomenon and injury pattern of the so-called “weekend warrior” [24–27].

Looking into the reported figures, one must remember that only data on absolute emergencies are somewhat reliable. Other conditions might be strongly influenced by the weekly rhythm, where it might, for example, be considered problematic to go to the ER on the weekend; thus, patients may stall until Monday. Another bias running in the opposite direction is that on weekends, the only institutions offering medical care to the general population are ERs, with private practices and smaller private hospitals being closed during these days. This conglomerate of factors gives rise to a phenomenon known as “the weekend effect” in the literature. In a meta-analysis of 68 studies encompassing over 640 million admissions, Chen and colleagues concluded that the weekend effect was significantly greater for elective than for emergency or maternity admissions [20]. While, on average, fewer patients are admitted to the hospital on weekends than during the week, those accepted are more severely ill [20].

When organising the infrastructure of the health care system, especially the ERs, these deliberations are of secondary interest. Of relevance for correct staffing in the ERs is, ultimately, the actual patient volume frequenting the installation to avoid overcrowding and impaired quality of care. In that respect, it can also be questioned whether data from older studies should still be considered, given that patient demographics, medical comorbidities of the population, understanding and treatment of most diseases, working conditions, and the infrastructure of the healthcare system have drastically changed over the past 50 years. Resource allocation should thus be based on locally generated data to serve present needs.

The strengths of the present study include the large dataset covering six years in a hospital of maximum care (level 3 trauma centre) and a systematic approach based on DRG- and OPS-codes, which reduces selection bias and prevents recall bias. Limitations are the lack of additional information that would allow for drawing inferences on risk behaviour, such as alcohol consumption, smoking or leisure time activities with a high potential for injuries. In a subset of diagnoses, such as ankle sprains or contusions, not all patients will report to an ER, thus preventing any conclusions concerning incidence or prevalence.

## Conclusion

Fluctuations of patient volume in ERs have been described for many different diagnoses. In musculoskeletal trauma, we observed a relevant increase in registered cases during the weekends in a tertiary centre of maximum care in Germany. While we cannot present data on the reasons for this increase, it is probable to account for this rise in a different activity profile of the patients compared to the work-week. Other factors that might influence this are the patients’ obligations during the work week and the availability of medical care limited to ERs on weekends. Independent of all these reasons, these data may help healthcare providers allocate their resources based on patient volume and emergency conditions.

## Supplementary Information

Below is the link to the electronic supplementary material.

Supplementary Material 1

## Data Availability

The datasets used and/or analysed during the current study are available from the corresponding author on reasonable request.
